# The Reliability of CT Scan Measurements of Pelvic Incidence in the Evaluation of Adult Spondylolisthesis

**DOI:** 10.7759/cureus.21696

**Published:** 2022-01-28

**Authors:** Jinhui Shi, Swamy Kurra, Michael Danaher, Frank Bailey, Katherine H Sullivan, William Lavelle

**Affiliations:** 1 Orthopedics, The First Affiliated Hospital of Soochow University, Suzhou, CHN; 2 Orthopedic Surgery, State University of New York Upstate Medical University, Syracuse, USA; 3 Orthopaedics and Rehabilitation, The University of Vermont, Burlington, USA; 4 Anesthesiology, University of Chicago Medicine, Chicago, USA

**Keywords:** lumbar lordosis, age, spinopelvic parameters, sacral slope, pelvic tilt, degenerative spondylolisthesis, isthmic spondylolisthesis, spondylolisthesis, computed tomography (ct), pelvic incidence (pi)

## Abstract

Background: Pelvic incidence (PI) has been described as a parameter that may be a risk factor for lumbar spondylolisthesis (SPL). Studies have reported PI measurement is more precise in CT scans. Very limited studies have measured PI using CT scans to evaluate SPL. We analyzed the reliability of CT scans to measure PI to evaluate SPL and compared it to patients without SPL.

Methods: A retrospective, cross-sectional study of PI in a consecutive cohort of patients’ pelvic/abdominal CT scans from an emergency room visit at a Level 1 trauma center between 2013 and 2016. Inclusion criteria was >18 years and had no lumbar or pelvis fracture. A total of 361 patients met the criteria for our study. We documented age, average PI, and SPL (type, grading, and location). Sagittal CT scans were used to measure PI (between hip axis to an orthogonal line originating at the center of superior end plate axis of first sacral vertebra). Patients were categorized: with SPL (n=45) and without SPL (n=316). Subgroups were comprised based on the location of SPL (L4/L5 and L5/S1) and type of SPL. Analysis of variance (ANOVA) and chi-square tests used; p≤0.05 considered statistically significant.

Results: Patients with SPL were significantly older versus patients without SPL, p=0.006. There were no statistical differences in PI between patients with and without SPL (p=0.29); between subgroups of patients with SPL at L4/L5 and without SPL (p=0.52); between subgroups with type of SPL at L4/L5 and without SPL (p=0.47); and between SPL patients at L5/S1 and without SPL (p=0.40). Patients with isthmic SPL at L5/S1 had nearly significant higher PIs (p=0.06) compared to those without SPL or with degenerative SPL at L5/S1. There was a trend towards higher PI in Grade 2 SPL patients at L5/S1, p=0.18.

Conclusions: Patients with SPL were significantly older than patients without SPL. The two trends observed were that PI was higher in patients with isthmic SPL at L5/S1 and an increased PI with Grade 2 isthmic SPL at L5/S1. Our reported CT PI measurements correlated with reported PI measured using standard radiographs in patients with SPL. CT scans may be a reliable modality to evaluate adult SPL.

## Introduction

When examining patients who have degenerative lumbar conditions, it is important to consider the potential for these conditions to develop during their lifetime. There has been significant research to identify patients with spinal conditions that develop in adolescence, such as spondylolysis and spondylolisthesis (SPL) [1,2]. Lumbar and pelvic radiographic parameters along with sagittal balance are well recognized as important predictive indicators for treating adult spinal deformity and SPL [3,4].

Spinopelvic parameters have been previously identified and reviewed in multiple studies [5-7]. These parameters include pelvic tilt (PT), sacral slope (SS) and pelvic incidence (PI) with PI being directly correlated to PT and SS [8]. PI is a key characteristic of the pelvis and unique to each person becoming set at the end of growth regardless of position. PI can vary between 33° to 85° in the normal population, with an average PI of 51.9° [9]. PI is defined as the angle formed between the line orthogonal to the sacral end plate and the line connecting the center of the sacral endplate with the center of the femoral heads [10,11]. PI has been described as a parameter that may be a risk factor for lumbar SPL [12]. Abnormal spinopelvic parameters may contribute to isthmic spondylolysis and degenerative SPL [8].

Sagittal alignment of the pelvis has usually been evaluated by two-dimensional sagittal radiographs in the standing position and pelvic parameters have been measured from these radiographs. While PT and SS vary in value based on the patient’s position, PI is an anatomical parameter because it remains the same regardless of the position of the patient. This allows for comparison between patients in the standing, sitting, and supine positions. Historically, PI measurements have been taken from lateral standing x-rays, but the superposition of the femoral head and mid-sagittal view of the pelvis and/or magnification can give false values.

Some studies have reported that measurement of PI is more precise in computed tomography (CT) scans [13]. While several studies have shown a correlation of PI with SPL through the use of radiographs, there have been limited studies using CT scans to measure PI in adult SPL patients [14-17].

The purpose of this study was to analyze if CT scans are a reliable modality in the measurement of PI for the evaluation of adult SPL. This article was presented at the Orthopaedic Research Society Annual Meeting as Poster #1712 on March 10-13, 2018 and as an electronic poster #19 for the 11th Lumbar Spine Research Society Annual Meeting on April 5-6, 2018.

## Materials and methods

This was a retrospective cross-sectional study of a consecutive cohort of patients undergoing a pelvic/abdominal CT scan from an emergency room visit at a Level 1 trauma center between 2013 and 2016. To be included in this study, the patient had to be greater than 18 years of age because PI increases during childhood and then remains unchanged throughout adolescence and adulthood [18,19]. We excluded patients who had sustained a lumbar or pelvis fracture. A total of 361 patients met the criteria and their records were reviewed and analyzed by measuring PI utilizing sagittal CT scans.

Measurement of PI in CT scans

**Figure 1:**
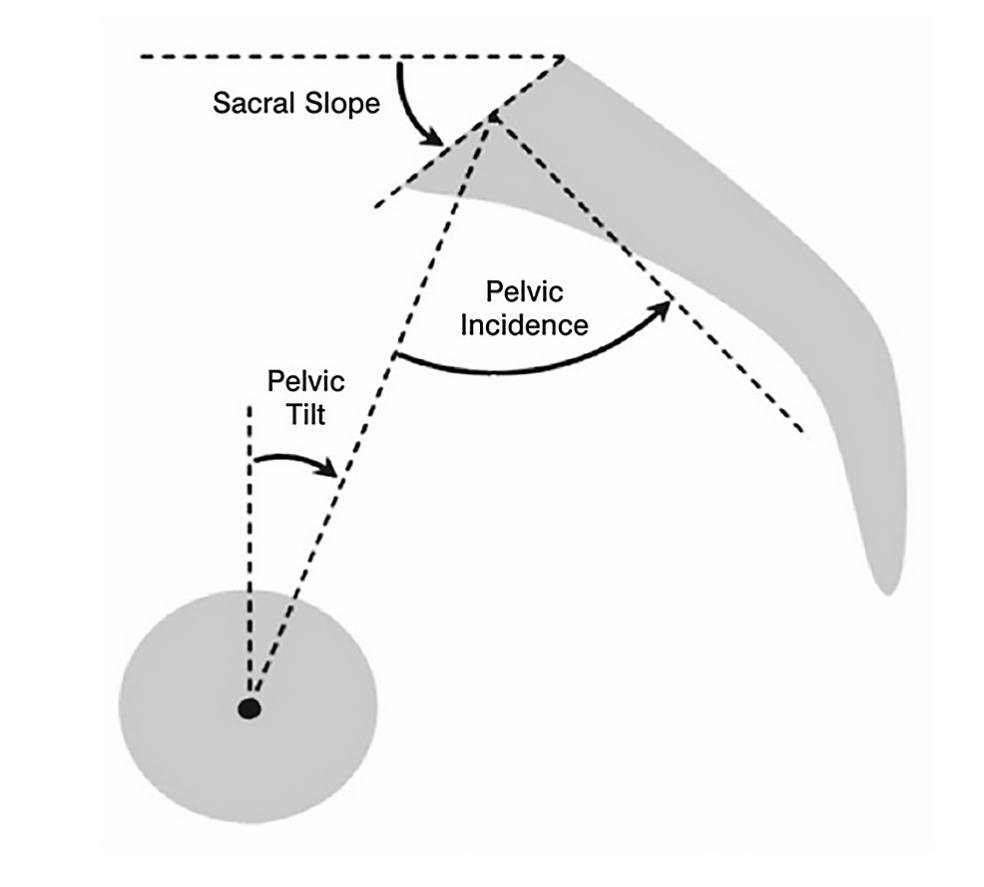
Illustration of how PI, PT, and SS angles are determined PI: pelvic incidence; PT: pelvic tilt; SS: sacral slope.

Supine lateral radiographs have been traditionally used to calculate PI [10,11]. Figure 1 illustrates how PI, PT, and SS are measured.

**Figure 2:**
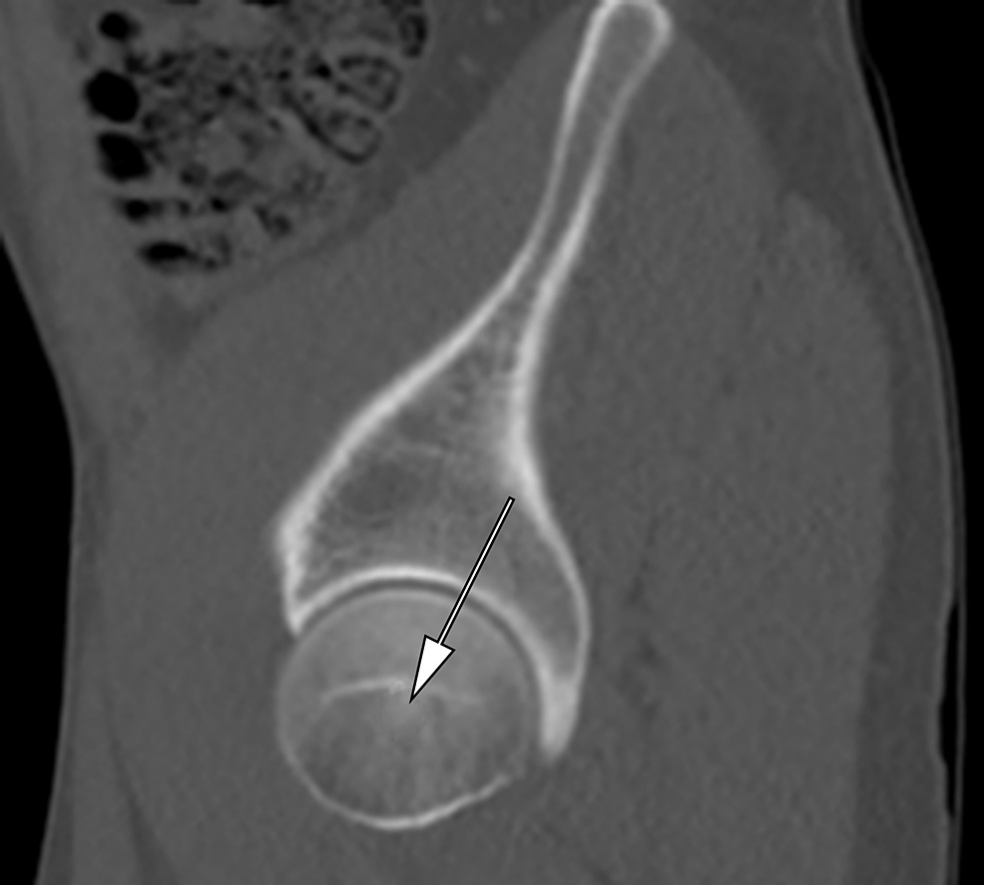
Place a mouse cursor at the center of the right femoral head and scroll the CT slices in sagittal view medially until the sacrum is seen

**Figure 3:**
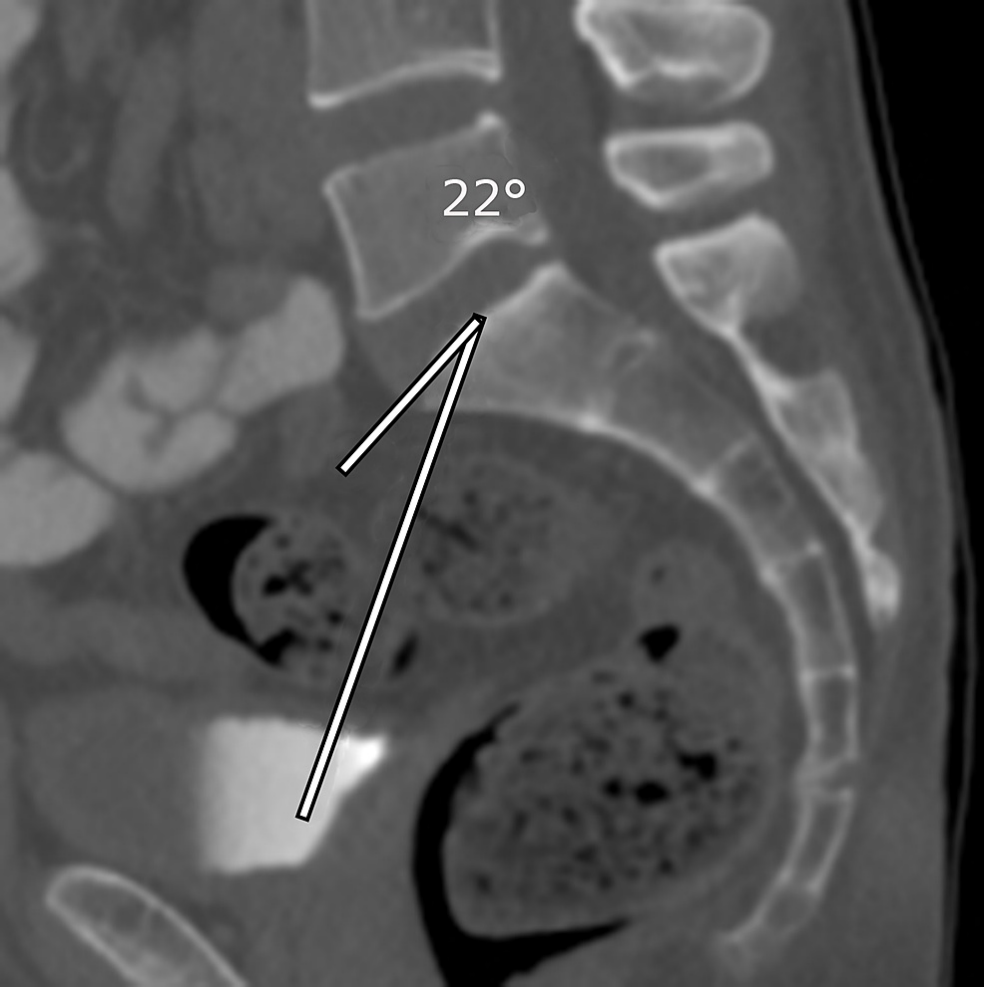
Draw a transecting line to the center of the sacral end plate from the center of the right femoral head (cursor). By subtracting the angle shown in the figures from 90°, the orthogonal angle was obtained to display the pelvic incidence (PI). For example, this patient’s right-sided PI = 90° -22°= 68°

For CT scans, to acquire the superposition of the two femoral heads seen on radiographs, we measured the PI of both the left and right femoral heads. The PI from both was averaged because PI can vary between each femoral head due to differences in the diameter of each femoral head. First, the center of the right femoral head is determined. Then, a line is drawn transecting the midpoint of the superior sacral end plate from the center of the right femoral head and draw another line down the slope of the superior sacral end plate from its midpoint. The angle formed between the two lines is subtracted from 90° to obtain the PI, right-sided PI = 90° -22°= 68° (Figures 2-3).

**Figure 4:**
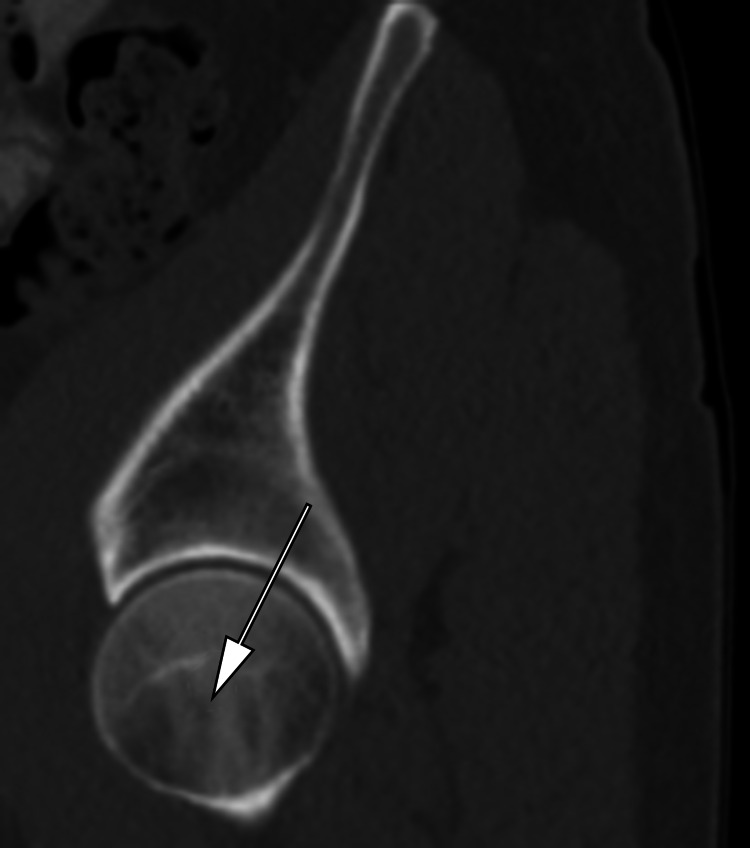
Place the mouse cursor at the center of the left femoral head and scroll the CT slices in sagittal view medially until the sacrum is seen

**Figure 5:**
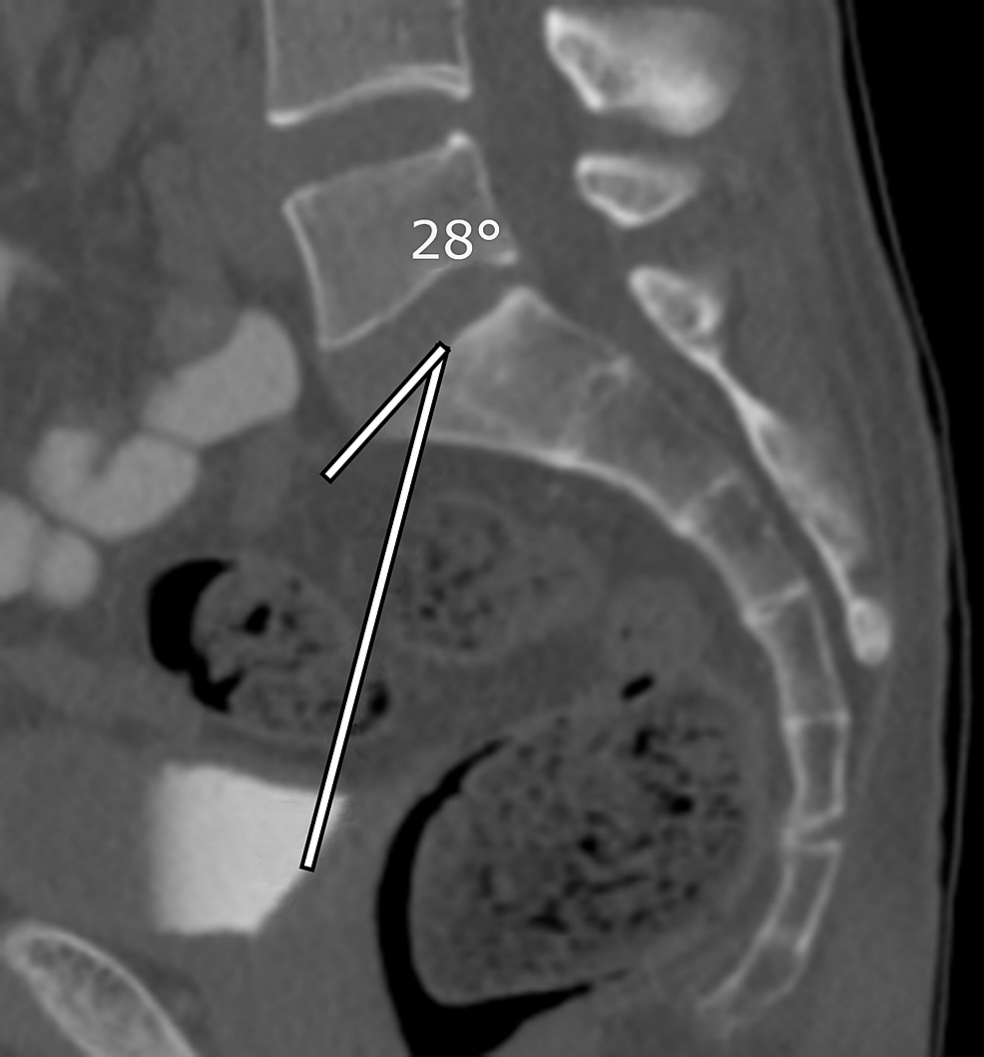
Draw a transecting line to the center of the sacral end plate from the center of the left femoral head (cursor). By subtracting the angle shown in the figures from 90°, the orthogonal angle was obtained to display the pelvic incidence (PI). For example, this patient’s left-sided sided PI = 90° -28° = 62° for the left-sided PI. Add the right- and left-sided PIs and divide by 2 to obtain the average PI = 68°+ 62°=65°

The same procedure was repeated for the left femoral head (Figures 4-5). The average PI was calculated using both left and right PI measurements. The average PI was calculated using both left and right PI measurements and dividing by 2. Averaging was used to adjust for obliquity in positioning.

**Figure 6:**
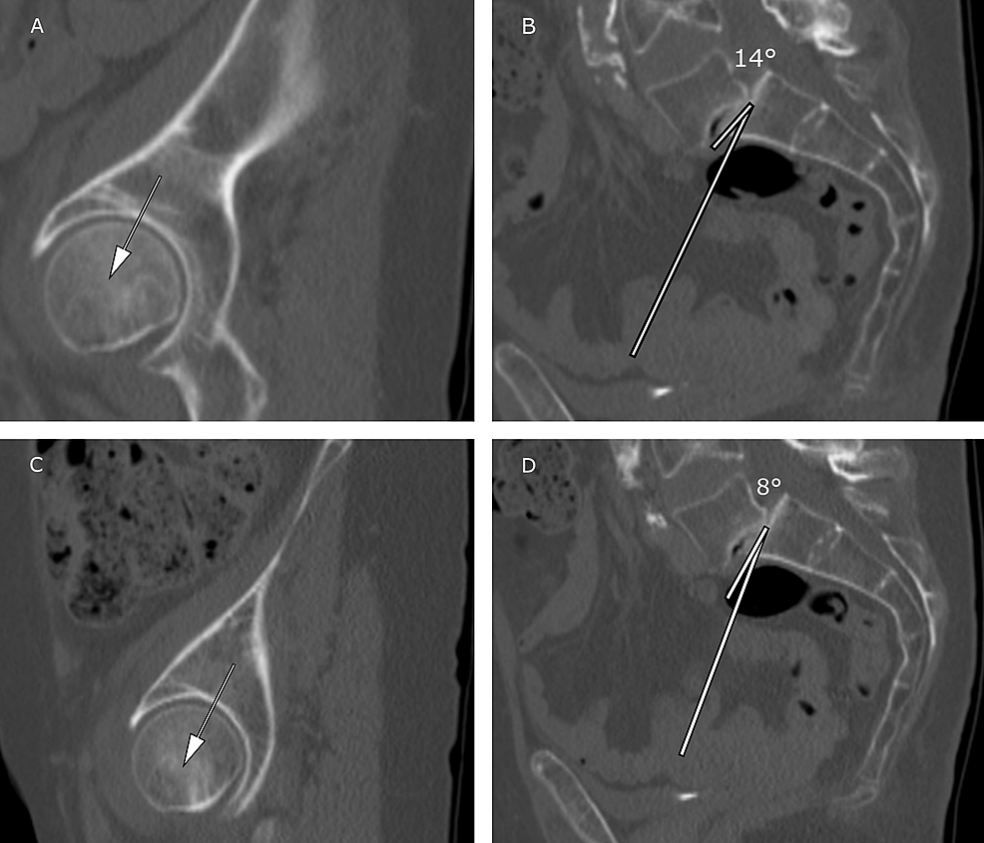
Patient with a Grade 2 spondylolisthesis of the L5 on S1. A) Illustrates locating the center of the right femoral head and B) depicts the determination of the right pelvic incidence (PI) angle, PI= 90°-14°=76°, C) Illustrates locating of the center of the left femoral head and D) depicts the determination of the left PI angle, PI= 90°-8°=82°, and average PI = 76°+82°/2 =79°

Figures 6A-6D detail the determination of PI measurements in a patient with Grade 2 SPL at L5/S1.

SPL grading was determined on the sagittal CT scans by using the Meyerding Classification System [20]. Patients were grouped based on those with SPL (n=46) and those without SPL (Grade 0) (n=315). We compared this data with age and PI. Subgroups were formed based on the location of the SPL (L4/L5 or L5/S1) and the type of SPL. Of note, only isthmic and degenerative SPL were observed in our data set. Isthmic SPL is the most common type of SPL and most frequently occurs at the L5-S1 level. Degenerative SPL is found in aging individuals and located most often in the lumbar spine. Congenital SPL, present at birth and rare; traumatic SPL, occurring after a large amount of force is exerted; pathologic SPL, associated with tumors, cancers, and bone disease; and iatrogenic SPL caused by prior surgery would not have been found in our inclusion data set. Age and PI were also analyzed among the subgroups.

Statistical methods

Statistical analyses were performed using SPSS version 25 (IBM Corp., Armonk, NY) software. For categorical data, a chi-square test was used; and for continuous measurements, an analysis of variance (ANOVA) test was used; p≤0.05 was considered as statistically significant.

## Results

Overall, the 361 patients involved in this study had a mean age of 58 years. Patients with SPL were significantly older versus patients without SPL, p=0.006. There was no statistical difference seen for PI between the two groups (Table 1). The prevalence of patients with SPL was 12.4% (46/361) in our study.

**Table 1:** Comparison of patients with and without spondylolisthesis

	Spondylolisthesis (L4/L5) and (L5/S1)	Without Spondylolisthesis	P value
n	46	315	
Mean Age in Years (range)	66 (30 - 95)	57 (21 - 97)	0.006
Pelvic Incidence Angle (range)	59° (34°- 80°)	57° (27° - 87°)	0.29

Based on the vertebral level, subgroups were defined as L4/L5 and L5/S1. The patient’s age and PI angles were compared between those with SPL and without SPL.

Table 2 summarizes the comparisons between the subgroup of patients with SPL at the L4/L5 vertebrae level versus patients without SPL. We documented 16 patients with SPL at the L4/L5 vertebrae level, 14 patients had a Grade 1 SPL (87.5%), and 2 patients had a Grade 2 SPL (12.5%). The mean age was significantly higher in patients with SPL at L4/L5, p=0.03. There was no statistical significance seen for PI between the two subgroups. Of the 16 patients, 8 patients had isthmic SPL, and 8 patients had degenerative SPL. On average, patients with degenerative SPL at L4/L5 were older than patients without SPL. No statistical significance was found with PI among subgroups

**Table 2:** Comparison of patients with spondylolisthesis at L4/L5 versus patients without spondylolisthesis

	n	Mean Age in Years (range)	Pelvic Incidence Angle (range)
Without Spondylolisthesis	315	57 (21 - 97)	57° (27° - 87°)
Spondylolisthesis (All)	16	69 (31 - 87)	59° (37° - 73°)
Isthmic Spondylolisthesis	8	66 (31 – 80)	61° (51° - 73°)
Degenerative Spondylolisthesis	8	71 (60 - 87)	56° (37° - 71°)
P value		0.03	0.52
		0.09	*0.47*

Another subanalysis was made between patients with SPL at the L5/S1 vertebrae level versus patients without SPL. Patients with SPL were statistically older versus patients without SPL (p=0.04). No significant difference for PI was noticed between patients. Patients with isthmic SPL at L5/S1 trended toward a higher PI (p=0.064). However, there was no significant difference for PI between the groups (Table 3).

**Table 3:** Comparison of patients with spondylolisthesis at L5/S1 versus patients without spondylolisthesis

	n	Mean Age in Years (range)	Pelvic Incidence Angle (range)
Without Spondylolisthesis	315	57 (21 - 97)	57° (27° - 87°)
Spondylolisthesis (All)	30	65 (30 - 95)	59° (34° - 80°)
Isthmic Spondylolisthesis	22	63 (30 - 95)	61° (34° - 80°)
Degenerative Spondylolisthesis	8	72 (48 - 94)	52° (41° - 72°)
P value		0.04	0.40
		*0.07*	0.064

Using the Meyerding Classification, of the 30 patients with SPL at L5/S1, 80% were Grade 1 SPL and 20% were Grade 2. There was a trend toward a higher PI in patients with Grade 2 SPL (L5/S1) but did not reach statistical significance (Table 4).

**Table 4:** Comparison between patients with Grade 1 or Grade 2 spondylolisthesis and patients without spondylolisthesis (Grade 0)

	Grade 0	Grade 1	Grade 2	P value
Mean Age (yrs.) (range)	57 (21 - 97) (n=315)	64 (30 - 95) (n=24)	69 (47- 86) (n=6)	0.10
Pelvic Incidence Angle (°) (range)	57° (27° - 87°)	57° (34° - 80°)	65° (57° - 73°)	0.18

## Discussion

In our evaluation of adult SPL utilizing CT scans to determine if they are a reliable modality, we measured PI because it has been established as a specific and constant value for every individual and represents the orientation of the pelvis [21]. Additionally, PI does not change with the patient’s position because the sacrum and pelvis together form a rigid structure [13,21]. The association between PI, SS, and, PT can be demonstrated as PI=SS + PT [17,21]. PI represents the anatomical configuration of the pelvis and sagittal balance regulation. Numerous studies have reported that a greater PI angle could predispose a patient to SPL [4,22,23].

One of the most difficult aspects of measuring PI is to determine the precise location of anatomical landmarks. A previous study by Kim et al. [24] showed that the reliability of measuring PI manually was less compared to measuring PI with CT due to the variability of selecting the appropriate anatomical landmarks in radiographs. Similarly, Dimar et al. [25] found relatively low agreement in manually measuring PI on radiographs among experienced surgeons with an intra- and inter-rater reliability of 0.69 (0.62-0.74) and 0.41 (0.36-0.45), respectively. As stated in the introduction, some studies suggest that CT scans may be more precise in the measurement of PI [13].

Because PI can vary significantly between each femoral head, we felt it was important to measure PI for both right and left femoral heads and obtain an average PI. Presently, there is no cutoff PI value that determines if a patient is at risk to develop SPL. A confounding factor is that PI can vary widely in the normal population (between 33° to 85°) [9]. This means each patient would need to be evaluated individually.

Vrtovec et al. [13] measured and analyzed PI in three-dimensional images in a normal population. Their mean PI was 47.1°± 10.0°. They also concluded that a statistically significant correlation was obtained between PI and age. By applying linear regression, the relationships between PI and age were evaluated to PI= 0.13 × age + 41.67. We believe the reason why PI in their study was smaller than our study may be the mean age of the patients (mean age: 41.5 years; range: 1-87) was younger than our study (mean age: 58 years; range: 21-97).

Peleg et al. [26] also reported a study of measurements in three-dimensions for PI with results of 57°± 13° which were similar to our study results of a mean PI in SPL patients of 59°, and without SPL of 57°. However, Peleg et al. measured PI for a relatively small number of subjects (n = 20).

Grannum et al. [27] reported a study describing the relationship between pelvic parameters (PI, PT, SS, and lumbar lordosis) and their association with a mobile SPL. The results showed sagittal pelvic parameters did not play a significant role in differentiating between mobile and non-mobile degenerative SPL at L4/L5 (PI (p = 0.409), PT (p = 0.476), SS (p = 0.785), lumbar lordosis (p = 0.695)). According to our study, PI showed no significant difference between patients with SPL and without SPL at L4/L5. We also found there were no statistical differences in PI with isthmic SPL (L4/L5), degenerative SPL (L4/L5) and without SPL. From an anatomical perspective, PI is measured on the bases of the sacrum and femoral head, but not using L4 and L5. Therefore, PI cannot predict L4/L5 SPL and its progression.

Min et al. [28] reported that PI values showed a significant difference between low-grade and high-grade SPL; and had a significant correlation with the dislocation level in all 51 patients with L5/S1 SPL in their study. Hanson et al. [29] investigated the correlation of PI with low- and high-grade isthmic SPL using radiographs. They concluded that PI was significantly higher in patients with low- and high-grade SPL compared with controls. Our study also found the mean PI was generally higher in patients with Grade 2 SPL at L5/S1 (mean=65°, range: 57°-73°) compared to Grade 1 SPL at L5/S1 (mean=57°, range: 34°-80°) and without SPL (mean=57°, range: 27°-87°), but our results did not reach statistical significance; p=0.18. The mean PI for low grade SPL (Meyerding 1 and 2) in the Hanson et al. study was 68.5° like our average PI in Meyerding 2 (65°). Similar to Hanson et al. [29], the adult control group PI (57°) was equal to our study’s PI value (57°) in patients without SPL. In our study, the mean PI trended toward a higher value in patients with isthmic SPL at L5/S1 (mean=61°) and lower in degenerative SPL at L5/S1 (mean=52°) compared to without SPL (mean=57°), although these findings did not reach statistical significance (p=0.06). The results demonstrated that PI could predispose a risk for SPL at L5/S1. Our study PI measurements using CT scans in SPL patients are similar to the SPL PI values reported in literature.

Some limitations of our study were its retrospective nature, and all patients were from one institution. The SPL subgroups were small and there may be a potential for sampling bias that limits interpretation of the results although it has been reported that 5% to 7% of the population has isthmic SPL. Additionally, Chakravarthy et al. reported the prevalence of degenerative SPL as 4.1-11.1% within the general population [30]. These estimates do fall within our study’s findings which observed 12.4% had either isthmic or degenerative SPL. Another limitation is CT scans may miss SPL due to the reduction of an unstable segment and, in some patients, SPL has only been evident on an upright radiograph of the lumbar spine. Studies with a larger sample size, a randomized design and a multicenter setting are required to determine the reliability of CT scans in the measurement of PI in adult SPL. 

## Conclusions

Two trends were observed: PI was almost significantly higher in patients with isthmic SPL at L5/S1; and there was an increased PI in patients with Grade 2 isthmic SPL also at L5/S1. Our reported PI from CT scans correlated with PI reported in literature using standard radiographs in patients with SPL at L5/S1. Therefore, CT scans may be a reliable tool to evaluate adult SPL and surgeons may be able to utilize PI values to assess the risk factors for developing SPL or other degenerative lumbar conditions.
